# Lattice Surgery for Dummies

**DOI:** 10.3390/s25061854

**Published:** 2025-03-17

**Authors:** Avimita Chatterjee, Subrata Das, Swaroop Ghosh

**Affiliations:** 1 Department of Computer Science & Engineering, The Pennsylvania State University, State College, PA 16801, USA; amc8313@psu.edu; 2 School of Electrical Engineering and Computer Science, The Pennsylvania State University, State College, PA 16802, USA; subratadas@psu.edu

**Keywords:** quantum error correction codes (QECCs), encoding, transversal gates, lattice surgery

## Abstract

Quantum error correction (QEC) plays a crucial role in correcting noise and paving the way for fault-tolerant quantum computing. This field has seen significant advancements, with new quantum error correction codes emerging regularly to address errors effectively. Among these, topological codes, particularly surface codes, stand out for their low error thresholds and feasibility for implementation in large-scale quantum computers. However, these codes are restricted to encoding a single qubit. Lattice surgery is crucial for enabling interactions among multiple encoded qubits or between the lattices of a surface code, ensuring that its sophisticated error-correcting features are maintained without significantly increasing the operational overhead. Lattice surgery is pivotal for scaling QECCs across more extensive quantum systems. Despite its critical importance, comprehending lattice surgery is challenging due to its inherent complexity, demanding a deep understanding of intricate quantum physics and mathematical concepts. This paper endeavors to demystify lattice surgery, making it accessible to those without a profound background in quantum physics or mathematics. This work explores surface codes, introduces the basics of lattice surgery, and demonstrates its application in building quantum gates and emulating multi-qubit circuits.

## 1. Introduction

Quantum computing leverages quantum mechanics principles to perform tasks unachievable by classical computing, with applications spanning molecule simulation for drug development, financial modeling improvements, machine learning advancements, optimization task enhancements, and supply chain management transformations [1,2,3,4,5]. Yet, the path to commercializing these innovations faces hurdles, including issues with qubit stability and quantum noise [6,7]. Quantum error correction codes (QECCs) play a pivotal role in realizing fault-tolerant quantum computing amidst inherent qubit noise [8,9]. Unlike conventional error correction approaches [10], quantum error correction encounters specific obstacles due to the no-cloning theorem [11] and the phenomenon of wave-function collapse during qubit measurement [12]. This ongoing research has yielded a variety of quantum codes, including five-qubit, Bacon–Shor, topological, surface, color, and heavy-hexagon codes [13,14,15,16,17,18], each contributing to the advancement towards fault-tolerant quantum computation.

### 1.1. Motivation

All QECCs are designed to encode a single qubit. When faced with a circuit involving multiple qubits that require QECC application, enabling interactions among these encoded qubits becomes necessary. QECCs typically employ transversal gates for this purpose, which offer scalability but increase gate overhead, significantly diminishing the QECCs’ error-correcting capabilities. This challenge is addressed by lattice surgery [19], a technique that enables interactions between multiple encoded qubits without compromising their error-correcting properties while keeping the increase in overhead manageable. Lattice surgery is essential to achieve fault-tolerant quantum computing, as it ensures QECCs can scale up to larger systems. Predominantly associated with surface codes, among the most prominent QECCs due to their low error thresholds and practicality for large-scale implementation, lattice surgery’s principles also lay the groundwork for advancing other QECCs [20]. Examples of such work include the qubit lattice surgery [21] and lattice surgery for color codes [20]. Understanding lattice surgery is thus crucial for developing fault-tolerant quantum computing.

### 1.2. Contribution

While grasping lattice surgery is vital, it presents significant challenges due to the sophisticated mathematical concepts of quantum physics, especially concerning surface codes and qubit interactions. The research papers on lattice surgery [22,23,24,25,26,27,28,29,30] often assume a deep understanding of such concepts, making it difficult for newcomers to follow. This work aims to offer a clear and approachable overview of lattice surgery’s key principles, tailored for researchers with limited experience in quantum physics or its mathematical underpinnings. This work provides a comprehensive overview of surface codes before delving into the fundamental principles of lattice surgery. It begins with the basics of lattice surgery, progresses through the construction of quantum gates using this technique, and ultimately demonstrates how lattice surgery can be used to emulate multi-qubit circuits. Prior familiarity with surface codes is not required for readers of this review. However, a basic acquaintance with quantum circuit symbols, as outlined in [31]—including fundamental measurement techniques, the controlled-NOT (CNOT) gate, and the Hadamard (H) gate—is presumed.

### 1.3. Paper Structure

The paper begins by laying a theoretical foundation on QECCs, surface codes, and transversal gates in Section 2. It then outlines the objectives of lattice surgery and its classifications in Section 3 and the range of operations achievable through lattice surgery in Section 4. The application of QECCs to circuits involving multiple qubits, facilitated by lattice surgery, is discussed in Section 5. Conclusions are drawn in Section 7.

## 2. Theoretical Background

### 2.1. A Brief Overview of QECCs

Quantum error-correcting codes (QECCs) are designed to protect quantum information from errors due to decoherence and other quantum noise, thereby enabling reliable quantum computation [9,32]. Understanding the interaction between physical and logical qubits, the encoding process, the types of errors encountered, and the use of stabilizers and ancilla qubits is fundamental in implementing QECCs effectively.

In quantum computing, physical qubits are the basic units of quantum information, analogous to bits in classical computing. These qubits can exist in states represented by superposition, allowing them to hold a combination of 0 and 1 simultaneously, and they can become entangled with each other, a property that underpins the power of quantum computing. Logical qubits, however, are formed by encoding quantum information across multiple physical qubits. This encoding uses redundancy to enhance fault tolerance against noise and errors [9,33]. Logical qubits are thus more stable constructs designed to implement the robust storage and manipulation of information in a noisy quantum environment. The encoding of quantum information into logical qubits involves distributing the state of a single qubit across a group of physical qubits. This distribution is managed through specific quantum gates that entangle the qubits in a way that any error affecting a single physical qubit can be detected and corrected without collapsing the overall quantum state. The robustness of a QECC is largely defined by how effectively it encodes and preserves the state of logical qubits despite errors in physical qubits.

Quantum computations contend primarily with two types of errors: (i) Bit-flip errors that flip the state of a qubit from |0⟩ to |1⟩ or vice versa. This type of error is analogous to the classical bit flip and can disrupt computations by altering the basic state of a qubit. (ii) Phase-flip errors that alter the relative phase between the basis states of a qubit. In a superposed state, this error can change the relative weighting of |0⟩ and |1⟩, leading to a loss of coherence in the quantum information. Stabilizers are operators used in QECCs to check for errors without measuring the qubits directly, which would otherwise collapse their quantum state. There are typically two types of stabilizers used. Z-type stabilizers detect bit-flip errors by checking for unexpected changes in the qubit states. X-type stabilizers are used to detect phase-flip errors by observing changes in the phase relationships between qubits.

Ancilla qubits are additional qubits included in a QECC to aid in the error detection process [33]. They do not hold quantum information themselves but are used to interact with logical qubits to extract error syndromes—a form of indirect measurement that indicates whether and where an error has occurred. During syndrome measurement, ancilla qubits are entangled with logical qubits and then measured. The outcome of these measurements is used to diagnose errors based on the known properties of the stabilizers. Through syndrome measurement, the QECC can determine not just the presence of an error but also its type and possible location (using a suitable decoding algorithm), enabling targeted error correction that restores the integrity of the quantum information without needing to observe the logical qubits directly. This process underscores the sophisticated interplay between encoding, error detection, decoding, and error correction that QECCs use to maintain the fidelity of quantum computations amidst the inherent fragility of quantum states.

### 2.2. Surface Codes

Surface codes are a significant advancement in the field of quantum error correction, distinguished by their two-dimensional lattice structure that contributes to their high error tolerance [34]. Originating from the concept of toric codes, surface codes are a type of topological quantum error-correcting code that effectively utilizes the arrangement of qubits on a grid to safeguard quantum information against common quantum errors, specifically bit-flip and phase-flip errors [17,34].

These codes employ a unique strategy involving ancilla qubits to monitor the integrity of the quantum state. These ancilla qubits are integral to performing X and Z stabilizer checks across the lattice, allowing for the detection of the two primary error types without disrupting the quantum state of the data qubits. The effectiveness of these stabilizers in diagnosing errors is a key feature of surface codes, leveraging Pauli operations to probe groups of qubits and thereby determine the presence and locations of errors.

In the domain of quantum computing, the n-distance surface code is depicted using an n×n lattice configuration, where each position, or ‘blob’, represents a qubit that is crucial to maintain the logical state of the system. The structure of this lattice ensures that every qubit is monitored by both X and Z stabilizers [35]. These stabilizers are essential for identifying phase and bit-flip errors, respectively, utilizing combinations of Pauli operators (X or Z) that act on specific subsets of qubits within the lattice. Should an error occur, it alters the outcome of the stabilizer checks linked to the affected qubit. By conducting these stabilizer checks, it becomes feasible to pinpoint the timing and location of any errors using a decoding algorithm [36]. Once the presence and position of errors are confirmed, they can be rectified by applying quantum gates to flip the affected qubits back to their intended states, thus restoring the integrity of the quantum information.

The architecture of surface codes can be implemented in two variations, unrotated and rotated, each with a distinct lattice structure that affects their error-correction capabilities. The unrotated version features a square lattice where data qubits reside on the edges, and each square (or plaquette) links to a Z-stabilizer, with X-stabilizers located at the vertices connecting the squares [35]. Figure 1① shows an unrotated surface code, where qubits represented by grey blobs are influenced by Z-stabilizers depicted as purple squares and X-stabilizers illustrated with orange lines.

Conversely, the rotated version uses a tilted lattice that alternates X and Z stabilizers in a checkerboard pattern, impacting the qubits at each box’s vertices [37]. The stabilizers engage with the qubits located at the vertices of each specific box. Figure 1② displays a rotated surface code where purple surfaces represent Z-stabilizers and orange surfaces indicate X-stabilizers. Together, these stabilizers interact with all the qubits depicted as grey blobs, which form the logical state. This rotated arrangement often provides a slightly higher error threshold and is simpler to implement, making it more advantageous for robust quantum error correction over long distances.

When examining the functionality of these stabilizers in a quantum circuit, there are typically two main methodologies employed to demonstrate their interaction with the qubits. Figure 1③ illustrates the creation of a Z-stabilizer using a combination of CZ and Hadamard gates. This method enhances the Z-stabilizer’s capability to effectively detect bit-flip errors through a sophisticated manipulation of the quantum state. The configuration affects an ancillary qubit, which is visually depicted as a purple blob within the diagrams. After the interaction facilitated by the CZ and Hadamard gates, the state of this ancillary qubit is measured to provide a syndrome measurement, denoted as S_z_. This measurement is crucial as it reflects any errors detected across the qubits that the Z-stabilizer governs, showcasing the effectiveness of this gate combination in maintaining the integrity of the quantum state.

Figure 1④ demonstrates the construction of an X-stabilizer through the use of CNOT and Hadamard gates. This arrangement is designed to detect phase-flip errors, employing these gates to adjust the quantum state of another ancillary qubit, represented by an orange blob. The influence of the CNOT and Hadamard gates projects the outcome of this stabilizer operation onto the ancillary qubit, altering its state. Subsequently, the modified state of the ancillary qubit is measured to ascertain the syndrome value S_x_. This setup not only allows for precise error detection but also ensures that the corrections needed can be accurately determined and applied to maintain the quantum system’s overall fidelity.

In all cases, the interaction between the stabilizers and the ancillary qubits is critical for detecting errors effectively without altering the logical state of the quantum system. These visual and operational details highlight the intricate and precise nature of error correction strategies employed in quantum computing, specifically within the framework of surface codes. Although surface codes offer substantial benefits in error correction, their implementation demands a considerable number of physical qubits and sophisticated control systems. These requirements pose substantial hurdles in the development of a fault-tolerant quantum computer.

### 2.3. Transversal Gates

Surface codes are designed around a two-dimensional nearest-neighbor (2DNN) architecture, where qubits are arranged to interact primarily with their direct neighbors. This configuration aligns well with the physical layout of qubits on quantum chips, simplifying quantum operations and minimizing error probabilities due to its unity with the quantum chip’s architecture. The inherent design of surface codes ensures that error correction is efficiently achieved through interactions among nearest-neighbor qubits. Despite this, there has been a proposal to execute multi-qubit gate operations transversally across different surface codes [14,38,39]. Simplifying the concept, if an initial circuit contains an *X* gate, implementing it transversally means applying *X* gates to every qubit in the lattice. Likewise, suppose the original circuit involves a CNOT gate between two qubits. In that case, the transversal approach necessitates executing several CNOT gates from all qubits in one encoded lattice to those in another. Figure 1⑤⑥ illustrates the application of 2-qubit interactions transversally from one lattice to another in both unrotated and rotated surface codes. For superconducting qubit systems, where qubits are arranged in a fixed 2D grid with nearest-neighbor connectivity, rotated surface codes are generally more efficient because they reduce the required number of qubits and simplify syndrome extraction operations. On the other hand, trapped-ion architectures, which allow for all-to-all connectivity, have more flexibility in choosing between surface code variants, though compact encodings still offer advantages in reducing decoherence effects. Neutral atom arrays and other scalable quantum architectures are also exploring the use of surface code-inspired LDPC codes, where selecting an optimal variant will depend on hardware-specific error rates and interaction constraints.

Nonetheless, the necessity for transversal two-qubit gates has historically rendered planar encoding impractical in several scenarios where physical qubits are limited to 2D configurations and can only engage in nearest-neighbor interactions. This limitation is particularly evident in systems such as quantum dots [40,41], superconducting qubits [42,43], trapped atoms [44], nitrogen-vacancy centers in diamonds [45], and certain ion trap setups [46,47]. Implementing transversal gates in a surface code can challenge the 2DNN structure for several reasons: ➊ Breaking Locality: To perform a transversal gate, one needs to apply operations across potentially distant qubits simultaneously. In a strict 2D lattice, this means reaching beyond immediate neighbors, which disrupts the locality principle inherent to surface codes. Maintaining only nearest-neighbor interactions is crucial for minimizing error rates and implementation complexity. ➋ Increased Error Propagation Risk: While transversal gates are designed to prevent error propagation within their definition, the act of physically implementing these gates in a surface code setting—where we might have to engage non-neighbor qubits—increases the risk of spreading errors. This contradicts the surface code’s principle of localizing errors for easier detection and correction. ➌ Implementation Complexity: The surface code’s error correction relies on inherently local measurements. Introducing transversal gates necessitates control and synchronization across a wider array of qubits, complicating the implementation and potentially introducing more points of failure, which can degrade the error correction capabilities of the surface codes. ➍ Limited Set of Transversal Gates: Not all quantum gates can be implemented transversally in a way that maintains the 2DNN structure of surface codes. This limitation means that some desired quantum operations cannot be performed without compromising the local interaction model, thus forcing a trade-off between the types of operations one can perform and the preservation of the 2DNN structure.

In conclusion, although transversal gates contribute to fault tolerance, their integration within surface codes disrupts the 2DNN architecture by requiring interactions beyond immediate neighbors. This necessity introduces complexities in physical implementation, elevates the potential for error spreading, and restricts the variety of operations that can be executed while adhering to the principle of locality. These issues have been addressed through the innovative approach of lattice surgery, which involves the strategic ‘cutting’ and ‘merging’ of code surfaces. This method effectively preserves the integrity of standard nearest-neighbor interactions and fault tolerance, offering a solution to the challenges posed by transversal gates within the framework of surface codes.

## 3. Lattice Surgery

### 3.1. Pivotal Idea

Lattice Surgery involves the strategic manipulation of a lattice’s structure to obtain specific outcomes, utilizing two fundamental techniques: merging and splitting. Merging two lattices entails combining them into one unified surface. This process is facilitated by the measurement of joint stabilizers along the surfaces’ edges as part of error correction cycles. The behavior of the operation varies based on the edges that are connected. Conversely, splitting a lattice separates one surface into two by severing joint stabilizers, thereby creating additional boundaries. The characteristics of the newly formed boundaries dictate the properties of the resultant states. Every surface code has a rough edge and a smooth edge. They describe the boundaries of a two-dimensional lattice that encodes qubits. Visually, the rough edges are identified by the explicit termination of qubits at the lattice boundary. While, the smooth edges are defined by the absence of qubit termination, implying a conceptual continuation of the lattice structure beyond its physical confines. Rough edges, through the termination of qubits, provide a structural basis for the application of X-stabilizers. Smooth edges, characterized by the non-termination of qubits, facilitate the deployment of Z-stabilizers. The configuration of these qubits about the lattice’s boundary defines the operational dynamics of the code, particularly in the correction of bit-flip and phase-flip errors.

Lattice surgery plays a crucial role in fault-tolerant quantum computing by enabling logical operations through local stabilizer measurements rather than direct transversal gates. Unlike transversal gate-based approaches, which often require additional SWAP operations to align qubits for interaction, lattice surgery relies solely on merging and splitting logical qubits within a stabilizer code framework, thereby minimizing the need for long-range interactions. This makes it particularly advantageous in architectures constrained to nearest-neighbor interactions, such as superconducting qubits and certain ion-trap implementations. By reducing the circuit depth and limiting the number of physical qubit interactions, lattice surgery lowers the probability of error accumulation, a critical factor in scalable quantum computation.

Most quantum error correction techniques, including lattice surgery, assume Markovian noise models, where errors occur independently at each time step. However, real quantum hardware often exhibits non-Markovian noise, where error processes are correlated across time [48]. This is particularly important for lattice surgery, as non-Markovian effects can reduce the effectiveness of repeated syndrome extraction by introducing memory effects that standard decoders do not account for [49]. Non-Markovian noise also affects logical error rates, requiring modifications to traditional threshold calculations in fault-tolerant quantum computing. Recent studies suggest that non-Markovian-aware decoders may be necessary to maintain high error correction efficiency [50]. As experimental quantum systems improve, better characterizations of these effects will be critical for optimizing lattice surgery operations.

### 3.2. Lattice Merging

**Figure 2:**
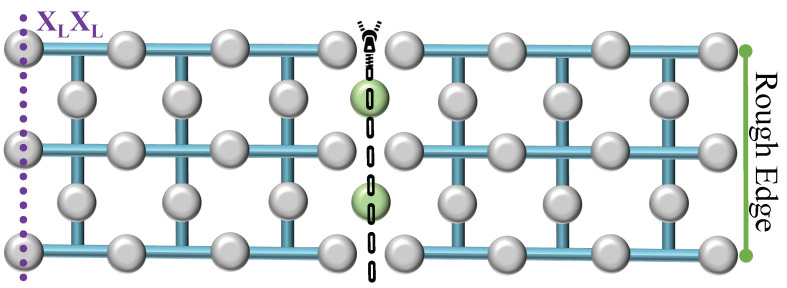
Rough surface merging. Two logical surface codes are unified by a row of transitional qubits (green blobs) initialized in the |0⟩ state, facilitating a merged surface encoded by the operation $X_{L}X_{L}$.

**Figure 3:**
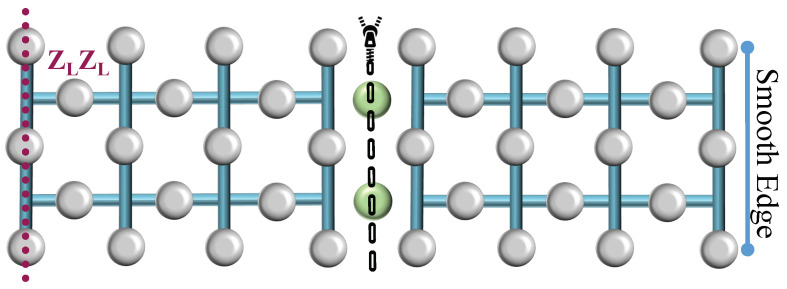
Smooth surface merging. The unification of two logical surface codes through transitional qubits (green blobs) prepared in the |+⟩ state, culminates into a merged surface characterized by $Z_{L}Z_{L}$.

Consider the setup depicted in Figure 2, comprising two logical surface codes, each encoding a single qubit, and a separate row of ‘transitional’ uninitialized physical qubits, marked in green. Initially, these qubits are set to the |0⟩ state. Subsequently, the two surfaces are unified by positioning the transitional qubits between them. Additional stabilizers are created around the new qubits to incorporate them within the system. The entire system then undergoes error correction as a unified data surface. The logical operation X, denoted as the old boundary, remains unchanged and is represented by the product of the logical operators from the two initial surfaces, thus denoted as $X_{L}X_{L}$. Consequently, this merging process, which combines the rough edges of the two surfaces, is identified as rough surface merging, resulting in a single, unified surface. In the second merging technique, referred to as smooth surface merging (Figure 3), the transitional qubits (depicted as green blobs) are initialized in the |+⟩ state. The resulting merge is characterized by the measurement of $Z_{L}Z_{L}$, distinguishing it from the rough surface merging by the initial state of the transitional qubits and the nature of the logical operation measured post-merge.

### 3.3. Lattice Splitting

**Figure 4:**
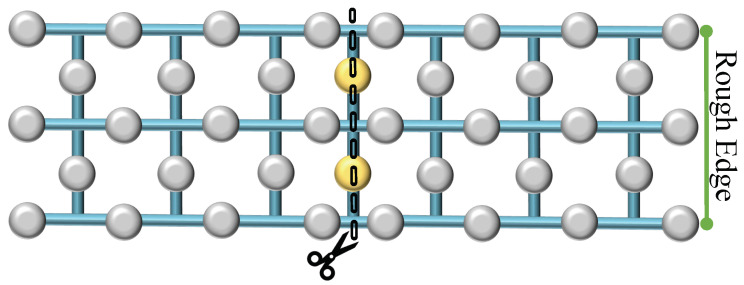
Rough surface splitting. Division of a single quantum surface into two separate entities via the measurement of transitional qubits (yellow blobs).

**Figure 5:**
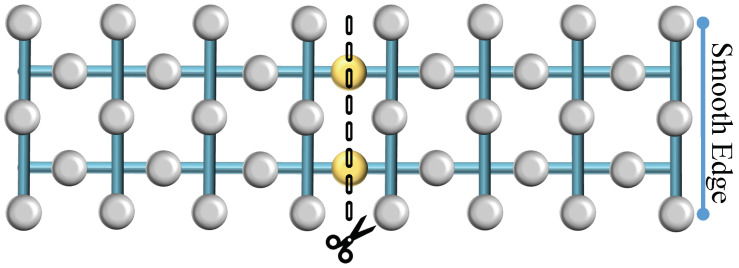
Rough surface splitting. The central row of qubits is measured to create separate surfaces with distinct boundary conditions.

Lattice splitting entails dividing a single surface into two by measuring a central row of qubits, effectively excising them from the lattice. This measurement results in the formation of two distinct surfaces upon completion of the operation. Similar to merging, splitting can occur along two types of boundaries: rough or smooth. In the case of a rough split, illustrated in Figure 4, the central row of qubits—visually denoted as yellow blobs—are measured in the Pauli-X basis, effectively partitioning the surface into two independent lattices. This division can alter the code’s distance, potentially affecting its error-correcting capabilities. Likewise, during a smooth split, as depicted in Figure 5, the central row of intermediate qubits—again represented as yellow blobs—is similarly measured out with a similar effect as a rough split.

## 4. Operations with Lattice Surgery

In this section, we describe the emulation of fundamental gate operations using lattice surgery. Familiarity with the logical *X* and *Z* behaviors is extended to examine additional gates.

### 4.1. The CNOT Gate

**Figure 6:**
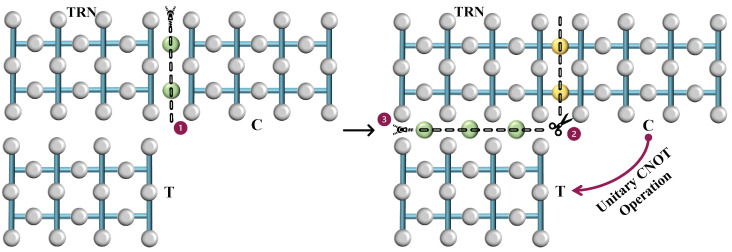
Implementation of the CNOT gate using lattice surgery. The process involves merging and splitting logical qubits *C* (control) and *T* (target) with a transitional surface TRN, strategically applying quantum operations to achieve the gate function within a surface code environment.

The CNOT gate creation process is a technique used to perform logical operations between qubits encoded in surface codes. The entire process is shown in Figure 6. We begin with two logical qubits of distance *d*, the control (*C*), and the target (*T*), each encoded on separate surfaces. The control is in an arbitrary state while the target is initialized in the state |+⟩. Just as ancilla qubits are utilized, we introduce an auxiliary surface known as the transitional logical surface (TRN). The TRN mirrors the structure of the surfaces of *C* and *T* and is, therefore, a surface with a distance *d*. This entire surface is initialized in the |+⟩ state at the start of the operation. This TRN surface is used to bridge the control and target qubits and facilitate the CNOT operation.

The first operation is a smooth merge between the surfaces *C* and TRN. The smooth merge is used here because it conserves the phase relationship, which is necessary for the control functionality of the CNOT gate. After the merge, a logical state dependent on the measurement outcome is formed. The merged surface is then split back into *C* and TRN. This split is also a smooth split. The purpose of the split is to ‘separate’ the control and the intermediary while preserving the state information transferred during the merge. Finally, the transitional TRN is merged with the target *T*. This merge effectively performs the controlled-NOT operation: if the control qubit was in the state |1⟩, the target’s state will be flipped. If the control was in |0⟩, the target remains unchanged. After the final merge between TRN and *T*, we achieve the CNOT operation between the control and target qubits. In the end, we are left with two logical qubits of distance *d*. The CNOT operation is also completely reversible.

The series of merges and splits are not redundant but rather a systematic way to transfer and manipulate the quantum information between the qubits to realize the CNOT gate. Each merge and split has a purpose: The first merge is to entangle the control qubit’s state with the intermediary. The split is necessary to retain the control’s state while preparing to apply its effect to the target. The second merge then applies the control qubit’s state to the target qubit, completing the CNOT operation. The process exploits the properties of quantum mechanics to implement computational logic in a fundamentally different way than classical logic gates. After completing this series of operations, the TRN becomes inactive, freeing the qubits for further use. We can either reinitialize the surface to the |+⟩ state for a forthcoming CNOT operation in the circuit or reset the qubits for a different purpose.

### 4.2. The Hadamard Gate

**Figure 7:**
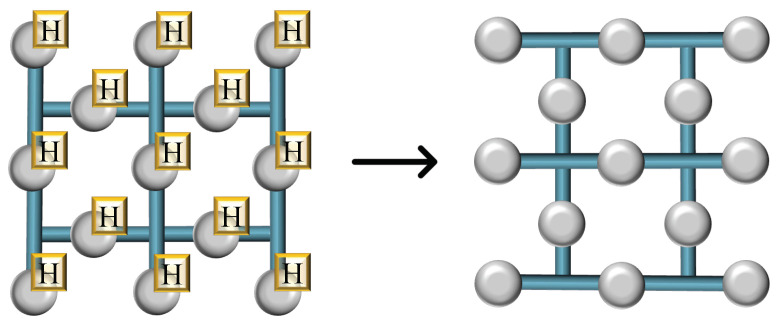
Orientation disruption post-transversal Hadamard gate. Resulting configuration after applying a transversal Hadamard gate: the individual *H* operations reorient the planar surface, diverging from its original layout as demonstrated in this figure.

Executing a Hadamard gate across an entire surface of qubits transversally through the application of the *H* operation to each qubit individually results in a planar surface that is correct state-wise. However, this approach alters the physical orientation of the planar surface relative to its initial configuration, as depicted in Figure 7. Such reorientation is acceptable if the qubits require no additional manipulation. Nonetheless, should subsequent operations be necessary, or if the preservation of the qubits’ interconnectivity is essential for scaling the system, an alternative strategy must be employed to realign the planar surface to its original layout.

**Figure 8:**
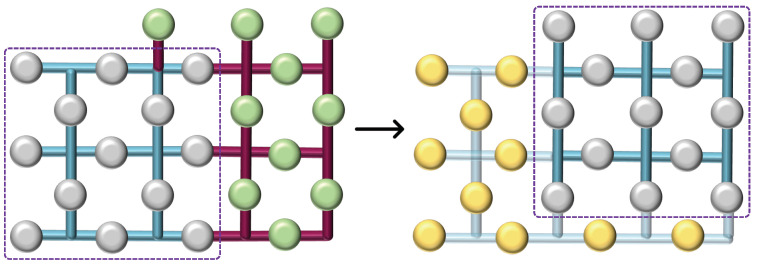
Restoration of planar surface orientation. Corrective process to realign the planar surface following a transversal Hadamard operation: Merging with auxiliary qubits and subsequent contraction through Z-basis measurements returns the surface to its original orientation, as depicted here.

To realign the planar surface post-Hadamard operation and preserve its original orientation—necessary for further quantum computations or to maintain the qubits’ connectivity for scalable quantum systems—specific corrective steps can be undertaken, as outlined in Figure 8. The process begins with the rotated surface, which is subsequently merged with additional qubits (depicted as green blobs). This merger creates an enlarged, stable surface, which compensates for the reorientation caused by the initial Hadamard operations. Subsequently, this expanded surface is methodically reduced back to its original size by measuring certain qubits (marked in yellow) in the Z-basis, effectively reversing the rotation. Through this series of splits, the planar surface’s alignment is restored. This corrective sequence of merges and splits functions due to the topological nature of surface codes, where logical operations like the Hadamard can be mimicked by altering the connectivity and layout of the qubits. The final outcome, after contracting the surface, is a qubit array that has effectively undergone a Hadamard transformation and is realigned to its initial configuration, ready for subsequent quantum processing steps.

### 4.3. Arbitrary Qubit Rotation Gates

Having grasped the principles of integrating CNOT and Hadamard gates within lattice structures, we can now extend our exploration to the technique of state injection across a whole lattice. Consider a quantum state Ψ=α|0⟩+β|1⟩, the application of an arbitrary quantum gate to this state effectively ‘rotates’ it, transitioning it to a new state $\Phi =\alpha^{\prime }|0\rangle +\beta^{\prime }|1\rangle$. This transformation is due to the fact that all quantum gates act as rotations in the state space. While transversal operations could be employed to achieve this effect, they would only result in an altered orientation of the lattice’s surface. To incorporate a specific arbitrary state into a given lattice, and to accomplish the desired quantum gate’s effect without altering the lattice orientation, we employ lattice surgery. This technique allows for the precise and controlled introduction of the rotated state into the quantum computing framework.

**Figure 9:**
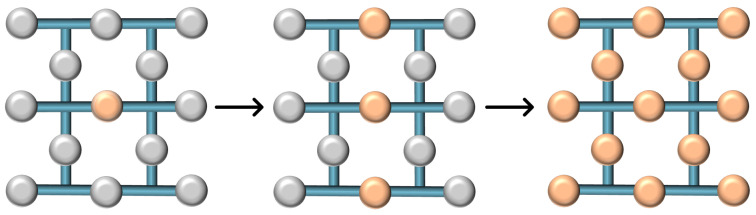
State injection into a quantum lattice. Sequential steps illustrating the injection of an arbitrary quantum state Φ into a lattice: starting with initialization, performing CNOT operations, swapping syndrome with data qubits, and spreading the state to achieve $\alpha^{\prime }{\left|0\right\rangle }_{L}+\beta^{\prime }{\left|1\right\rangle }_{L}$.

The procedure for state injection is illustrated in Figure 9. To inject a state Φ into a lattice, we begin with all qubits in the lattice initialized to the state |0⟩, except for one, which is in the state Φ. As shown in the first panel of the diagram, the grey qubits represent those in the state |0⟩, and the orange qubit is in the state Φ. CNOT operations are conducted between the orange qubit and the adjacent syndrome qubits. These syndrome qubits are subsequently swapped with the neighboring data qubits to create a three-qubit state $\alpha^{\prime }|000\rangle +\beta^{\prime }|111\rangle$, as indicated in the second panel of Figure 9. The lattice undergoes routine stabilizer operations, effectively spreading the encoded state across the entire structure, as depicted in the third step of Figure 9. This distribution is facilitated by syndrome measurements, which function as repeated applications of CNOT gates. This series of operations culminates in the injection of the state onto the entire lattice, through progressive entanglement, yielding a new logical state $\alpha^{\prime }{\left|0\right\rangle }_{L}+\beta^{\prime }{\left|1\right\rangle }_{L}$.

**Figure 10:**
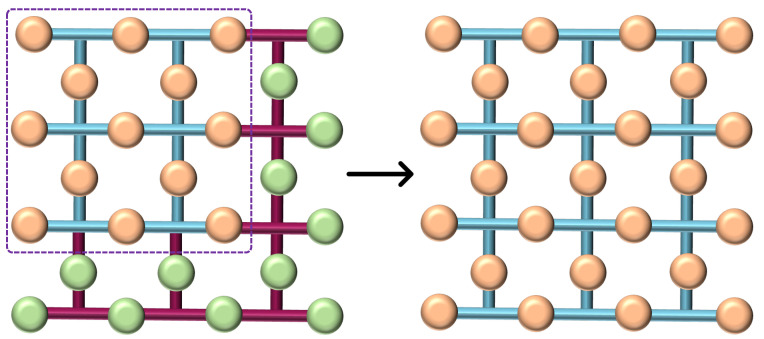
Scaling the injected state in a higher distance lattice. The process of expanding an injected state across a larger lattice to form a high-distance logical state, depicted through the inclusion of additional |0⟩ qubits and stabilization to reach $\alpha^{\prime }{\left|0\right\rangle }_{L^{\prime }}+\beta^{\prime }{\left|1\right\rangle }_{L^{\prime }}$.

Let us imagine a single qubit influenced by one rotational gate, $R_{x}\left(\theta \right)$. This qubit is allocated its own dedicated surface. We then select a single qubit from this surface and apply the same rotational gate, $R_{x}\left(\theta \right)$, as used on the original qubit. Following the procedure outlined in Figure 9, we proceed with the stabilizer operations or CNOT gates until the entire surface replicates the effect of the rotational X gate on the qubit. Once an injected surface is established, it can be scaled up to a surface with a higher distance. This scalable approach extends the injected state Φ across a larger lattice to achieve a higher error-correcting distance. As illustrated in Figure 10, the expansion of the injected state onto a broader lattice is facilitated through the incorporation of extra qubits (marked in green) initialized in the state |0⟩. Subsequent rounds of stabilizer measurements are then performed, allowing the state Φ to permeate the enlarged lattice. This process culminates in the formation of an augmented logical state, represented as $\alpha^{\prime }{\left|0\right\rangle }_{L^{\prime }}+\beta^{\prime }{\left|1\right\rangle }_{L^{\prime }}$, across the new, higher distance surface.

## 5. Application of QECC on Multi-Qubit Circuits

### 5.1. Moving Towards Fault-Tolerant Quantum Computing

We have explored how lattice surgery facilitates quantum operations, focusing primarily on single-qubit tasks and extending up to two-qubit computations, such as implementing a CNOT gate between two-qubit lattices. However, as quantum computing scales up to include circuits with a larger number of qubits, the complexity of applications also grows, demanding numerous qubits and gates. To achieve fault tolerance in these expansive applications, utilizing multiple QECC surfaces, each corresponding to individual qubits within the circuit is necessary.

**Figure 11:**
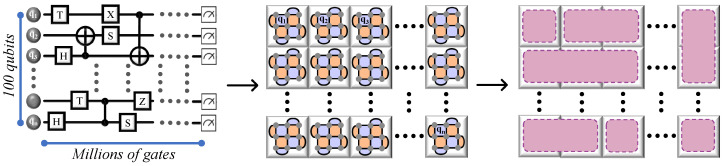
Integration of QECC in large-scale quantum circuits. Depiction of a complex quantum circuit comprising 100 qubits, each represented by individual surface codes in a tiled layout. This diagram illustrates how each tile (or surface) interacts with others through operations like merging, splitting, expanding, and contracting to faithfully emulate the gate operations of the original quantum circuit, ensuring fault tolerance in large-scale applications.

This multi-surface approach is just the beginning. There is also a critical need to orchestrate interactions among these QECC surfaces to accurately replicate the gate operations found in the original quantum circuit. Figure 11 illustrates an arbitrary quantum circuit with 100 qubits, encompassing millions of gates. Here, each qubit is represented by a distinct tile-like surface code, where each tile corresponds to a logical surface associated with specific qubits. The interaction between these tiles—merging, splitting, expanding, or contracting—is crucial for emulating the intended gate operations within the circuit, ensuring that the larger-scale quantum computing applications remain robust and error-resistant. Executing a quantum circuit on a surface code-based quantum computer as efficiently as possible involves an optimization challenge. This challenge, which has been proven to be NP-hard [28], focuses on minimizing both the number of surface code tiles and the number of time steps required to implement quantum algorithms. The objective is to achieve the most efficient use of resources in terms of both space and time. While the NP-hard nature of optimizing lattice surgery operations presents a significant challenge, there are additional technical and theoretical obstacles that must be addressed to achieve large-scale quantum computing. One such issue is hardware error accumulation, particularly in systems where stabilizer measurements are imperfect. Decoherence and correlated noise can lead to errors that are more difficult to correct, requiring robust adaptive decoding strategies [26]. Another major challenge is the classical computational overhead associated with real-time syndrome decoding. As lattice surgery scales up, the speed of classical error correction must keep pace with quantum operations. Recent works suggest that machine-learning-assisted decoders may provide a scalable solution to this bottleneck [51]. Furthermore, fault-tolerant state injection remains a critical issue, as lattice surgery alone does not provide a universal gate set—requiring high-fidelity magic state distillation, which can introduce significant operational overhead [24].

In practical implementations, qubit noise and operational errors can affect the stability of lattice surgery operations. To mitigate these issues, multiple error suppression techniques are employed. The most fundamental method is repeated stabilizer measurements, which help identify errors before they propagate to logical qubits. Additionally, advanced decoding algorithms such as minimum-weight perfect matching (MWPM) and belief propagation decoders allow for more efficient correction of errors caused by environmental noise. Furthermore, correlated noise models, which account for time-dependent and spatially correlated errors, are increasingly being integrated into hardware-level optimizations. Addressing these issues is essential for scaling lattice surgery in real-world quantum devices.

In comparison to ion-trap and superconducting quantum computing platforms, lattice surgery exhibits specific advantages. Ion-trap architectures allow long-range gates, which theoretically reduce the need for nearest-neighbor constraints; however, they suffer from slower gate speeds and increased decoherence times, making error correction cycles more challenging. Superconducting qubits, on the other hand, are typically arranged in a 2D grid layout, where long-range interactions are restricted, making lattice surgery a natural choice for executing fault-tolerant logical operations efficiently. While both ion-trap and superconducting approaches can implement logical operations using other techniques, lattice surgery fits seamlessly within stabilizer-based quantum error correction and offers a scalable and hardware-compatible method for performing logical operations in these systems.

### 5.2. Simplification of the Circuit

**Figure 12:**
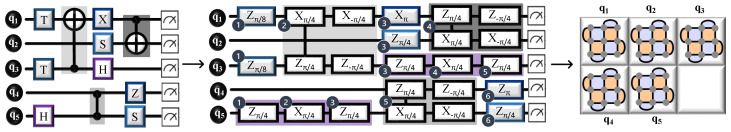
Quantum circuit decomposition and surface code mapping. Illustration of the process of decomposing a complex quantum circuit (first circuit) into its Pauli rotational forms (second circuit) and the subsequent integration with lattice surgery through surface codes (third diagram). Each qubit in the circuit is associated with a surface code, shown here as distance 3 rotated codes.

One effective method to sequentially execute all interactions from the original circuit is to decompose them into simpler subproblems. We recognize that each quantum gate can be expressed in terms of a Pauli rotation, denoted as $P_{\phi }=e^{-iP\phi }$, where *P* represents a Pauli product operator such as *Z*, *X*, *Y*, or Y⊗X, among others, and ϕ is an angle [24]. Table 1 provides a list of quantum gates along with their corresponding forms in Pauli rotations. By utilizing these rotational forms, we can simplify a quantum circuit, which facilitates the application of techniques like lattice surgery later on. To illustrate how lattice surgery can be effectively integrated into a quantum circuit, let us examine a specific example, as depicted in Figure 12. This example circuit comprises various single-qubit and multi-qubit gates. The initial step involves decomposing all the circuit’s gates into their Pauli rotational forms. This decomposition is crucial as it facilitates the injection of quantum states into the appropriate surface codes where needed. The decomposed version of this circuit is presented in Figure 12. Since the circuit involves five qubits, there will correspondingly be five surface codes, each linked to one of the qubits. The surface codes illustrated here are distance three rotated codes, although the specific type of code used can vary based on the physical error characteristics of the quantum system.

**Figure 13:**
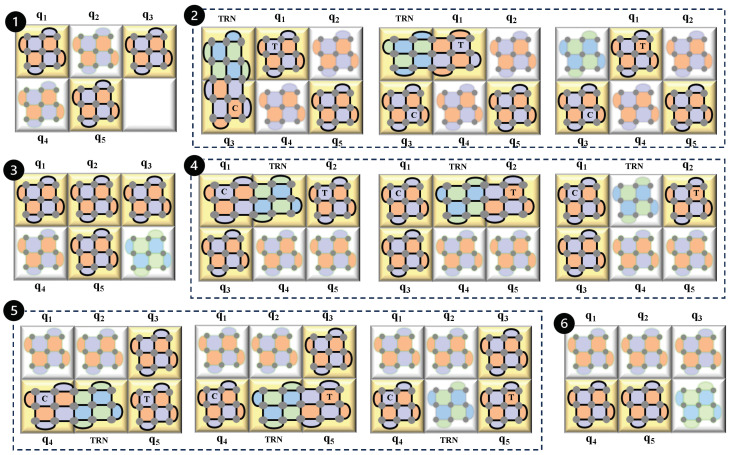
Detailed steps of lattice surgery in a quantum circuit. This figure demonstrates the step-by-step integration of lattice surgery techniques in a decomposed quantum circuit (second circuit of Figure 12). Starting with state injections for the *T* and *H* gates in the first step, the diagram progresses through the creation of CNOT and CZ gates and concludes with logical operations on specific qubits. Each step corresponds to the placement of state injections and logical gate operations, illustrating how lattice surgery manipulates and measures stabilizers to maintain the fidelity of the quantum state throughout the process.

After decomposing the circuit, we describe lattice surgery, which is a six-step process, as showcased in Figure 13. At each stage of the process, the yellow tiles indicate the active surfaces involved in the operation of that specific step, while the other blurred surfaces represent those that are inactive during that step. The first step starts with a state injection into the qubit, $q_{1}$ and $q_{2}$ for the *T* gates and another state injection into qubit $q_{5}$ for the first decomposed gate of the *H* gate. The second step creates the CNOT gate between the lattices of qubits $q_{1}$ and $q_{3}$. For this, we need to initialize a transitional lattice marked as TRN. To emulate the CNOT gate, the TRN is initially merged with qubit $q_{3}$. Subsequently, they are separated, allowing the TRN to then merge with qubit $q_{1}$. In the final arrangement, $q_{3}$ serves as the control and $q_{1}$ as the target, while the TRN becomes inactive. Although the TRN qubits can be repurposed for other uses, in this example, we will reinitialize the TRN surface to the |+⟩ state each time it becomes inactive. This ensures that the surface is ready to function as a TRN surface for subsequent multi-qubit operations. The third step contains four substeps—the first applies a logical *X* gate on the lattice of the qubit, $q_{1}$, then it proceeds to three state injections—into the lattice of $q_{2}$ for the *S* gate, then into $q_{3}$ for the first decomposed gate of the *H* gate and lastly on $q_{5}$ for the last decomposed gate of the *H* gate. The fourth step mimics the CNOT gate between the lattices of qubits, $q_{1}$ and $q_{2}$, by using a transitional lattice marked as TRN, followed by a state injection into $q_{3}$ for the second decomposed gate of the *H* gate. To simulate the CNOT gate, the TRN first merges with qubit $q_{1}$. After this, the TRN and $q_{1}$ are separated, which then allows the TRN to merge with qubit $q_{2}$. In the concluding setup, $q_{1}$ acts as the control qubit and $q_{2}$ as the target, while the TRN becomes inactive. The fifth step establishes a CZ gate between the lattices of $q_{4}$ and $q_{5}$ making use of the transitional lattice TRN. To replicate the CZ gate, the TRN initially combines with qubit $q_{4}$. They are then separated, enabling the TRN to subsequently merge with qubit $q_{5}$. In this final configuration, TRN is rendered inactive. This step also involves a state injection on the qubit, $q_{3}$, for the last decomposed gate of the *H* gate. The final or sixth step involves applying a logical *Z* operator onto qubit $q_{4}$ and a final state injection onto qubit $q_{5}$ for the last *S* gate. With this, we finish all the steps of lattice surgery and the final stabilizers after all sorts of merging, splitting, expanding, and contracting, which can be measured and to mimic the true nature of the original five-qubit circuit.

Lattice surgery, as depicted for smaller quantum circuits, scales up to encompass systems with hundreds of qubits, marking a critical progression towards fault-tolerant quantum computing. In large-scale implementations, lattice surgery involves the intricate manipulation of a vast network of qubits, each encoded and interconnected through surface codes or other error-correcting codes to form a robust quantum lattice. By extending the principles observed in smaller setups—such as sequential state injections, logical operations mapped across multiple qubits, and adaptive merging and splitting of stabilizers—lattice surgery on hundreds of qubits allows for complex computational tasks that are beyond the reach of classical computers. The modular nature of lattice surgery aids in efficiently managing quantum resources, enabling selective entanglement and disentanglement of qubits as required by the algorithmic demands. Furthermore, the ability to execute error correction through merging and splitting surface code patches without the need to physically relocate qubits or disrupt the entire quantum state highlights the practicality of lattice surgery in large-scale applications. A key advantage of lattice surgery is its logical-level circuit simplification. While any quantum gate can be decomposed into a sequence of rotation gates or controlled operations, this decomposition does not necessarily lead to efficient fault-tolerant implementations. A Hadamard gate, for example, typically requires three rotations when expressed in terms of native quantum gates. However, in lattice surgery, a logical Hadamard can be implemented through a topological transformation of the logical qubit, avoiding the need for additional physical gate applications. This significantly reduces the physical error accumulation that occurs with direct gate decompositions. More generally, logical operations in lattice surgery are performed through stabilizer reconfiguration rather than gate-based operations, leading to a net reduction in overhead in large-scale computations.

## 6. Feasibility and Implementation of Lattice Surgery

Lattice surgery has been experimentally explored and theoretically expanded across various quantum computing implementations. While the foundational surface code lattice surgery approach is widely used for enabling logical qubit interactions through merging and splitting operations [19], several advanced implementations have emerged to enhance its efficiency and applicability across different quantum hardware architectures. One such extension is twist-free lattice surgery, which avoids the need for twist defects within the lattice, thereby simplifying gate scheduling and circuit complexity [27]. Similarly, temporally encoded lattice surgery reduces algorithm run times by leveraging time-domain encoding techniques without requiring additional physical resources [27]. These approaches make lattice surgery more adaptable to a broader range of hardware implementations beyond traditional superconducting qubit layouts.

Beyond standard surface codes, subsystem lattice surgery (SLS) allows for fault-tolerant quantum information transfer between different topological codes, using only two-body nearest-neighbor interactions, making it a promising method for hybrid error correction architectures [52]. Additionally, compiler-based implementations of lattice surgery, such as the Lattice Surgery Compiler, have been developed to efficiently translate logical quantum circuits into lattice surgery operations, facilitating their execution on real quantum processors [53].

Several experimental efforts have validated the feasibility of lattice surgery in real quantum hardware. Ion-trap quantum processors have been used to successfully demonstrate lattice surgery, enabling entanglement between logical qubits and logical state teleportation. This experiment provides a crucial validation of lattice surgery in practical quantum computing setups [22]. In another demonstration, Riverlane and Rigetti conducted experiments with superconducting qubits, focusing on the merge stage of lattice surgery, where logical qubits are combined into a larger logical qubit and then split apart. This experiment aims to analyze how decoders respond in real hardware conditions, providing insights into the practical implementation of lattice surgery for quantum error correction [54,55].

Further developments in experimental lattice surgery are taking place through collaborative initiatives such as the SurgeonQ project by IQM, Riverlane, and Zurich Instruments. This project is developing a quantum error correction platform that includes lattice surgery experiments, with the goal of operating on logical qubits encoded from multiple physical qubits. The work being done under this initiative demonstrates the potential for large-scale quantum error correction using lattice surgery techniques [56,57,58]. These experimental efforts reinforce lattice surgery as a viable approach to fault-tolerant quantum computing, bridging the gap between theoretical methods and real-world quantum processors. As quantum hardware continues to evolve, these experiments will play a key role in refining scalability, error suppression, and decoding algorithms for practical large-scale quantum computation.

## 7. Conclusions

This paper attempts to simplify the complex topic of lattice surgery in the field of fault-tolerant quantum computing. It explores surface codes and demonstrates the practical application of lattice surgery in constructing quantum gates and simulating multi-qubit circuits to address this complex subject. As quantum computing scales to encompass larger and more complex systems, lattice surgery provides a critical framework, thereby enhancing the robustness and operational capabilities of quantum computers. The continued evolution of this field will likely see lattice surgery not only adapting to new quantum architectures but also inspiring analogous methodologies across various error-correcting paradigms, which will be crucial for achieving scalable and fault-tolerant quantum computing.

## Figures and Tables

**Figure 1:**
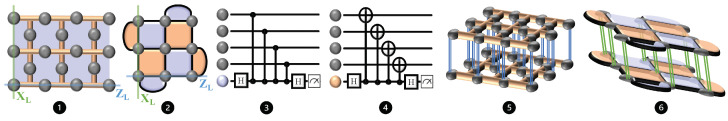
Surface codes. ① Structure of an unrotated surface code. ②: Structure of an rotated surface code. ③: Circuit representation of a Z-stabilizer acting on four qubits and projecting its syndrome onto an ancilla qubit, which is later measured. ④: Circuit representation of an X-stabilizer acting on four qubits and projecting its syndrome onto an ancilla qubit, which is later measured. ⑤: Visualization of transversal 2-qubit gates between two unrotated surfaces. ⑥: Illustration of transversal 2-qubit gates between two rotated surfaces.

**Table 1:** Quantum gates and their Pauli rotational form.

Quantum Gates		Rotational Form
**Single** **Qubit**	**X**	$X_{\pi }$
	**Y**	$Y_{\pi }$
	**Z**	$Y_{\pi }$
	**RX**	$X_{\theta }$
	**RY**	$Y_{\theta }$
	**RZ**	$Z_{\theta }$
	**H**	$Z_{\pi /4}\cdot X_{\pi /4}\cdot Z_{\pi /4}$
	**S**	$Z_{\pi /4}$
	**T**	$Z_{\pi /8}$
**Multi** **Qubit**	**CNOT**	${\left(Z⊗X\right)}_{\pi /4}\cdot {\left(1⊗X\right)}_{-\pi /4}\cdot {\left(Z⊗1\right)}_{-\pi /4}$
	**C(P1, P2)**	${\left(P_{1}⊗P_{2}\right)}_{\pi /4}\cdot {\left(1⊗P_{1}\right)}_{-\pi /4}\cdot {\left(P_{2}⊗1\right)}_{-\pi /4}$

## Data Availability

No new data were created or analyzed in this study. Data sharing is not applicable to this article.
